# Advances on Photonic Crystal Fiber Sensors and Applications

**DOI:** 10.3390/s19081892

**Published:** 2019-04-21

**Authors:** Vincenza Portosi, Dario Laneve, Mario Christian Falconi, Francesco Prudenzano

**Affiliations:** Dipartimento di Ingegneria Elettrica e dell’Informazione, Politecnico di Bari, Via E. Orabona 4, 70125 Bari, Italy; vincenza.portosi@poliba.it (V.P.); dario.laneve@poliba.it (D.L.); mariochristian.falconi@poliba.it (M.C.F.)

**Keywords:** laser, optical fiber, microstructured optical fibers

## Abstract

In this review paper some recent advances on optical sensors based on photonic crystal fibres are reported. The different strategies successfully applied in order to obtain feasible and reliable monitoring systems in several application fields, including medicine, biology, environment sustainability, communications systems are highlighted. Emphasis is given to the exploitation of integrated systems and/or single elements based on photonic crystal fibers employing Bragg gratings (FBGs), long period gratings (LPGs), interferometers, plasmon propagation, off-set spliced fibers, evanescent field and hollow core geometries. Examples of recent optical fiber sensors for the measurement of strain, temperature, displacement, air flow, pressure, liquid-level, magnetic field, and hydrocarbon detection are briefly described.

## 1. Introduction

Different kinds of optical fiber sensors are nowadays available on market at relatively low cost. These kinds of devices have become commercially interesting being integrated with practical detection and signal-processing electronics. However, innovative optical fiber sensors promise ever increasing numbers of features in the measurement of a large variety of physical parameters. Therefore, a huge volume of scientific literature has been produced on this topic during the last decades. Several laboratory prototypes have been constructed and characterized, paving the way for novel feasible measurement set-ups. Since the application of optical fiber sensors is extremely wide, an ever-increasing interest is fed by the needs of industrial, medical, military and civil areas. As examples, in mechanics to measure rotation, vibration, acceleration, bending, torsion, displacement, strain; in environmental monitoring to measure temperature, pressure, gas, chemical contaminants; in biomedicine and medical diagnosis to detect biomolecules and compound concentrations in biological fluids. Well-known strong points of optical fiber sensors are their compactness, which allows their housing in mechanical parts of a large variety of devices and systems without affecting their operation, the minimum weight, the immunity to electromagnetic interference, the high sensitivity [1,2]. It is well known that photonic crystal fibers (PCFs), thanks to their microstructured section have allowed the improvement of optical amplification and lasing, beam quality, high power delivering, extreme core confinement such as large mode area, nonlinear applications, group velocity dispersion control. Thus, these properties have allowed an improvement of optical fiber sensors, too.

The aim of this review is to provide a few recent examples of the potential of PCF sensors. It is not exhaustive due to the very large literature production. The focus is on: (i) plasmonic [3,4,5,6,7,8,9,10,11,12,13,14], (ii) interferometric [15,16,17,18,19] and (iii) grating-based [20,21,22,23,24,25,26,27,28,29,30,31,32] PCF sensors, discussed in the following Section 2, Section 3 and Section 4, respectively. In other cases, innovative materials as graphene, polyvinyl alcohol, ferrofluid liquids, combined with the exploitation of conventional principles as the interferometry or the evanescent field interaction are simultaneously applied to obtain novel and promising devices. A few of these last cases, coated PCF sensors [33,34,35,36,37,38,39,40,41,42,43,44,45,46,47], are briefly recalled in Section 5 of the paper. It is worthwhile to underline that the attempt to divide the state of the art of the sensors into well-defined sections is not trivial since these devices very often exploit a combination of operation principles. Also, the choice of the examples is very hard due to the high number of intriguing solutions reported in literature. In Section 6 the conclusions include a table recalling the main characteristics of the considered sensors, organized by taking into account the detected physical parameter and the operation principle.

## 2. Plasmonic PCF Sensors

Plasmonic fiber optic sensors exploit the phenomenon of Surface Plasmon Resonance (SPR) that can occur on the dielectric-metal interface of a thin film metal when the guided propagation mode couples with Surface Plasmon Polariton (SPP) mode under phase-matching conditions. When the light wavelength is the suitable one for the excitation of the plasmonic resonance, the Surface Plasmon Wave (SPW) on the metal adsorbs most of the energy carried by the incident light and a peak in the loss spectrum at the resonance wavelength can occur. The SPR condition is very sensitive to changes of refractive index (RI) of the dielectric neighboring the metal. In other words, a change of the optical property of the dielectric close to the metal, due to a variation of temperature, solution concentration, applied stress or strain, refractive index, etc, produce changes in the resonance wavelength. Consequently, a shift of the loss peak can be measured. The development of fiber optic sensors based on SPR is very promising because of the obtainable sensing characteristics, real-time detection, label-free biosensing.

In recent literature, SPR sensors have been designed and fabricated for measurement of refractive index of liquids [3,4,5,6,7,8,9,10], temperature [11,12], magnetic field [13], biomolecules concentration [14] for applications such as bio-sensing, environmental monitoring and medical diagnostics.

Plasmonic fiber optic sensors generally consist of an optical system to excite the plasmon resonance and a dielectric-metal system that transduces the variations of the quantity of interest in changes of RI. This can be detected by an optoelectronic system as a shift of the resonant wavelength. The performances of this sensors, sensitivity and resolution, depend by the characteristics of both optical system and the transducing medium. The metal layer is of nanometric thickness and is fabricated by deposition of a noble metal like silver [5,7,8,13] or gold [3,4,6,9,10,11,12,14] that have resonant peaks at the optical wavelengths. The optical part consists in an optical fiber suitably modified so that the evanescent field of guided mode and the plasmonic wave are coupled and the SPR is excited. The plasmonic medium is deposited within the fiber [4] or on its the external surface [5,6,9,10,12,14]. In the former case, the metal can coat the internal wall of some air-holes and these channels are filled by the liquid to be measured. In the latter case the fiber optic sensor is totally immersed in the analyte and the sensing region is the cladding and/or external surface. The first technique is more complex, from the fabrication point of view, but allows larger sensitivity. Other authors have proposed SPR sensors with metallic nanowires or nanorods instead of thin films [3,8,11]. The goal is to obtain a sensor that at the same time has a high sensitivity and a simple manufacturing process. Several optical fiber solutions have been investigated for the fabrication of SPR sensors like side-polished fibers [5,8,10] multimode fibers [7], no-core fibers [13] and PCFs [3,4,5,6,9,10,11,12,14].

PCFs, if compared with other solutions, exhibit the advantage of allowing an easy handling of the fiber optical dispersion by appropriately designing the sizes and positions of the air-holes, in order to favor the phase matching and coupling between the leaky core mode and SPP mode. Plasmonic PCFs are also very interesting due to their versatility, allowing monomodal behavior, high non-linearity and highly controllable birefringence.

**Figure 1:**
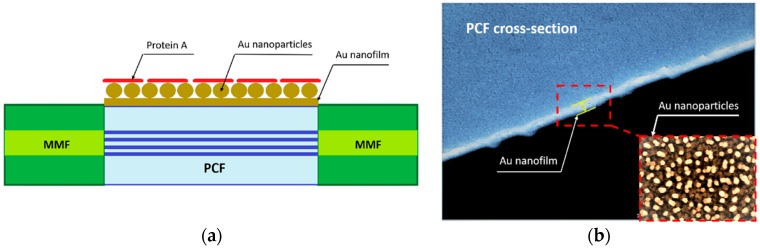
(**a**) Schematic of the SPR PCF sensor proposed in [14]. (**b**) SEM image of gold nanofilm on the PCF surface. Adapted from [14].

In [14] a plasmonic PCF sensor coated with protein A-gold nanoparticles-gold film was designed and experimentally investigated for the detection of immunoglobulin G (IgG), a type of human antibody. In this sensor, the coupling between the SPR on the gold-film surface and the Localized Surface Plasmon Resonance (LSPR) of ad-hoc located gold nanoparticles is employed to enhance the resonant plasmonic effect. The sensitivity is further increased by using the protein A co-modified as ligand of the IgG. In Figure 1a the sensor is schematically depicted. It consists of an MMF-PCF-MMF structure. The sensing region is coated by the thin gold film with gold nanoparticles and protein A. Figure 1b shows the SEM image of a part of the PCF cross-section and a zoom of the gold film with the gold nanoparticles.

**Figure 2:**
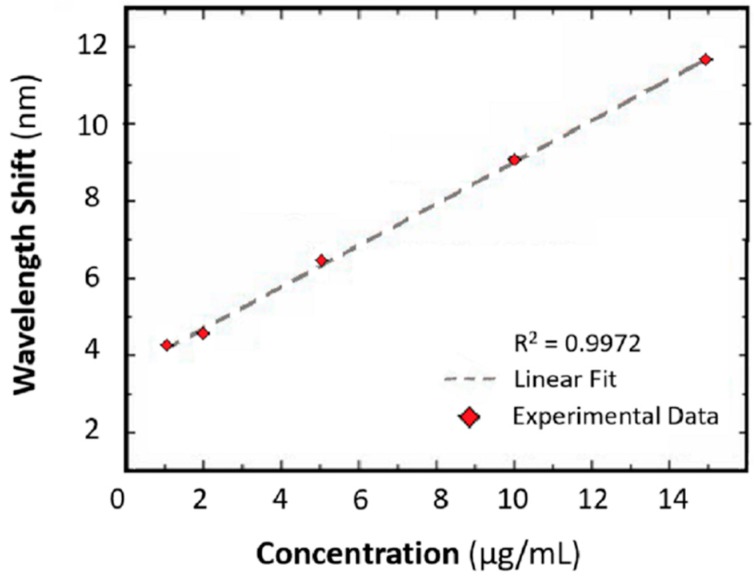
Wavelength red shift as function of IgG concentration. Adapted from [14].

The sensing performances are simulated and experimentally validated. The resonance wavelength shift versus the concentrations of human IgG, in the range 1–15 μg/mL, is measured and compared. Three types of SPR PCF sensors are considered: coated by Au film, Au nanoparticles-Au film and Protein A, Au nanoparticles-Au film, respectively. A refractive index (RI) sensitivity of 3915 nm/RIU and a resolution of 37 ng/mL are obtained; these values are 1.6 and 6.3 times larger than those of the Au-PCF sensor, respectively. In Figure 2 the wavelength shift as function of IgG concentration is illustrated and a good linear fit is observed. A sensitivity close to 0.54 nm/(μg/mL) and a coefficient of determination of 0.9972 are measured.

**Figure 3:**
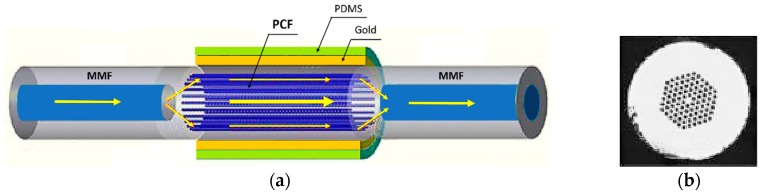
(**a**) Lay-out of the SPR optic sensor based on MMF-PCF-MMF structure. (**b**) Image of the cross section of the fabricated PCF-SPR sensor. Adapted from [12].

Another interesting plasmonic PCF optic sensor for temperature measurement has been presented in [12]. The optical system to excite the plasmonic resonance consists of a cascade of three fibers: multimode fiber—photonic crystal fiber—multimode fiber (MMF-PCF-MMF), shown in Figure 3a. An optical microscopic image of the fabricated sensor is shown in Figure 3b. As schematically represented in Figure 3a, the sensing area consists of a single mode PCF covered by a thin gold film of 60 nm thick and a polydimethylsiloxane (PDMS) layer whose refractive index varies sensibly with temperature due to its high thermal coefficient. The PCF core is smaller than that of MMF, thus most of the light that pass from MMF to PCF propagates in the PCF cladding to excite the SPR.

**Figure 4:**
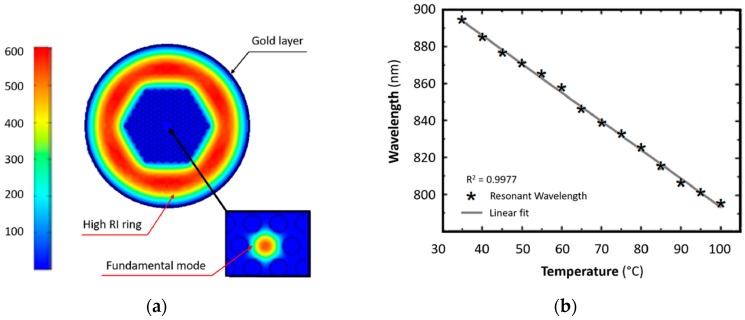
(**a**) Simulated mode field of PCF (**b**) Wavelength resonance as function of temperature. Adapted from [12].

Figure 4a shows the simulated PCF mode field. The cladding around the air holes of the PCF has a high refractive index so the high order cladding modes are confined near to the gold plasmonic layer and the SPR is strongly excited.

The fabrication of this kind of sensors is rather simple. The two ends of the photonic crystal fiber are soldered to the multimode fiber by an optical fiber splicer and the gold film is deposited by magnetron sputtering. Then the mixture of PDMS and curing agent is put on the sensing area and heated at 80 °C for two hours.

The sensing performances have been simulated and validated by experimental measurement. Solutions with different concentrations of glycerol, corresponding to refractive index changes from 1.3330 to 1.3904. The transmittance spectra have shown a red shift of 166.38 nm, by increasing RI from 1.333 to 1.3904. The maximum wavelength sensitivity of 4613.73 nm/RIU has been obtained.

The sensor has been placed in a temperature-controlled chamber to measure the variations of the transmittance spectra with temperature. A wavelength blue shift of 99.40 nm has been observed for the temperature change from 35 °C to 100 °C. In Figure 4b the resonant wavelength as function of the temperature is reported and a good linearity can be observed. A temperature sensitivity of −1.551 nm/°C has been measured.

**Figure 5:**
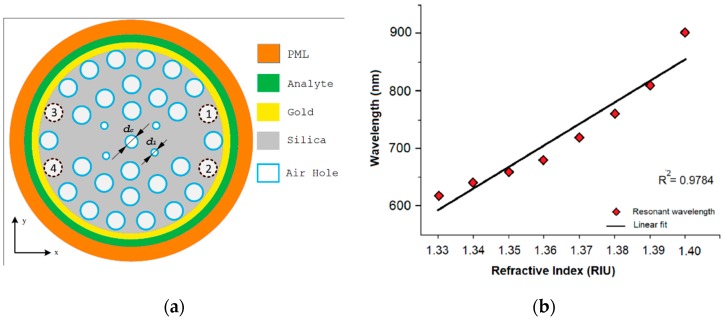
(**a**) Schematic of the PCF based SPR refractive index sensor proposed in [6]. (**b**) The fitting line of the simulated resonance wavelength versus refractive index of the analyte in the range 1.33–1.40 RIU. Adapted from [6].

As an example, to maximize the coupling a dual core PCF for a SPR refractive index sensor has been proposed in [6], as shown in Figure 5a. The distance between the metal surface and the core is reduced and the evanescent field can more easily reach the interface dielectric-metal to excite plasmon resonance.

The dual core PCF has three rings of air-holes which are arranged in a hexagonal and circular lattice respectively in the first two and third rings. The two cores are located on the opposite side of the central hole (*d_c_* in Figure 5a), in the regions without holes, i.e. by exploiting a total internal reflection mechanism (TIR). The parameters of the structure have been optimized at the wavelength of 0.62 μm for an analyte with refractive index of 1.33. A gold layer of 40 nm of thickness is deposited on the external surface of the fiber and the device is totally immersed in the analyte. The performances of the sensor have been evaluated by simulations for x- and y-polarized core guided modes and for variations of refractive index from 1.33 to 1.40, in steps of 0.01. The wavelength and amplitude sensitivity, resolution and linearity have been compared for the refractive indexes considered for both polarizations. Figure 5b shows the resonance wavelength as function of the analyte RIs.

Maximum amplitude sensitivities of about 725.89 RIU^−1^ and 1085 RIU^−1^ have been calculated, respectively, for x- and y-polarization. The maximum wavelength sensitivity is 9000 nm/RIU and the average wavelength sensitivity is 4000 nm/RIU for both polarizations. The maximum wavelength resolution corresponds to 1.11 × 10^−5^ RIU, while the coefficient of determination (R^2^) is 0.9784. Therefore, the simulations promise the possibility to obtain high performance SPR PCF sensors. 

An alternative strategy for plasmonic-based fiber sensors is to consider the sensing area of the SPR sensors based on a D-shaped photonic crystal fiber, a PCF with partly removed cladding on which a metal thin film is deposited, so that the surface plasmonic resonance can be excited. The flatness of the surface has the advantage with respect to the circular fiber surface of ensuring a larger uniformity of the thickness of the deposited metal layer with simpler manufacturing processes. 

**Figure 6:**
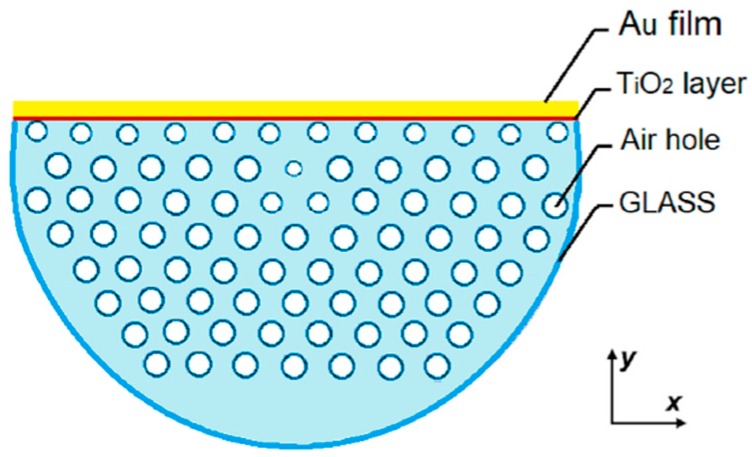
Schematic of the cross section of the D-shaped PCF SPR sensor. Adapted from [10].

An example of D-shaped PCF SPR sensor has been proposed in [10] and the schematic is shown in Figure 6a, where a layer of TiO_2_/A_u_ has been deposited on the exposed section of a microstructured fiber. The thin layer of TiO_2_, placed under the layer of plasmonic metal in gold, helps the adhesion of the metal on the silica of the fiber. The TiO_2_ is transparent at the wavelength of interest, so the light propagation is not significantly affected and, for an adequately thin thickness, the coupling with SPP mode is not hindered. The thicknesses of the TiO_2_ and Au layers have been analytically optimized by comparing the loss spectra and amplitude sensitivity by varying the thickness. The optimized structure has a TiO_2_ layer and a gold film of 5 nm and 40 nm of thickness, respectively. The PCF has a side polished for a depth of 8 μm. To improve the sensor performance, a birefringent effect has been obtained by scaling down the air holes of the first ring, with the exception of the two holes located on the sides of the core. The y-polarized TE mode has been considered due to the stronger coupling between the evanescent field of guided mode and the plasmonic wave. The analyte is placed in contact on the top of the gold film surface.

The loss spectra have been calculated at various analyte RIs in range from 1.33 to 1.43 and wavelength sensitivity, amplitude sensitivity and resolution have been estimated. Maximum sensitivity of 1086 RIU^−1^ and resolution of 9.2 × 10^−6^ RIU have been calculated using the amplitude interrogation method. The maximum and average sensitivity of nm/RIU and 9800 nm/RIU and a resolution of 2.2 × 10^−6^ have been calculated using the wavelength method. A prototype of the D-shaped PCF has been fabricated by the stack-and-draw method.

## 3. Interferometric PCF Sensors

A wide class of interferometric photonic crystal fiber (PCF) sensors are based on the idea of using the core and the cladding of a single-core PCF as the sensing and the reference arm of an interferometer, respectively [15,16,17]. As evidenced in the introduction, this operation principle is also exploited in other sensors which could be classified by considering a different criterion as the employed material of the kind of light matter interaction, see the last case of previous section. In dual- or multi-core PCFs, the interference between the fundamental modes of the different cores are exploited as well as the interference between the fundamental modes of one core and its higher order modes [18,19]. In sensing applications involving PCFs, the length of the PCF determines the length of the interferometer. When a change is induced in the effective refractive index of the modes propagating in the sensing arm of the interferometer, such sensing modes undergoes a phase change that leads to a change in the optical path difference with the modes propagating in the reference arm of the interferometer. As a consequence, a wavelength shift in the transmission spectrum (interference fringes) can be observed.

**Figure 7:**
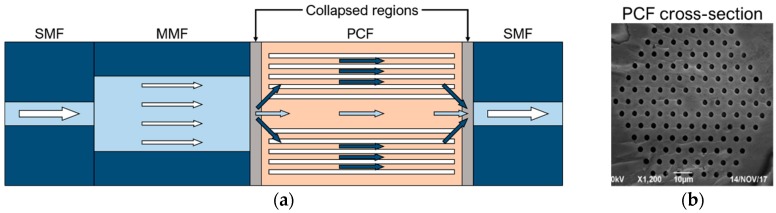
(**a**) All-fiber Mach-Zehnder interferometer strain sensor based on an in-line SMF-MMF-PCF-SMF structure; (**b**) Cross-section of the PCF. Adapted from [15].

**Figure 8:**
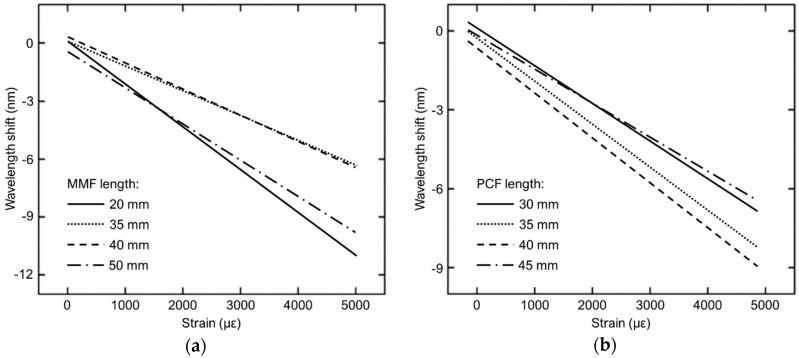
Measured wavelength shift of the MZI sensor response as a function of increasing applied strains for (**a**) different lengths of the MMF section (PCF length: 40 mm) and (**b**) different lengths of the PCF section (MMF length: 30 mm) [15].

In [15] an all-fiber in-line Mach-Zehnder interferometer (MZI) strain sensor is experimentally demonstrated. As shown in Figure 7, the MZI sensor is fabricated with a 50/125 μm multimode fiber (MMF) and a 10.1/125 μm photonic crystal fiber (PCF) spliced between two identical single mode fibers (SMFs) working in the C+L optical band (1528 ÷ 1602 nm). At the MMF-PCF interface, only a fraction of the power transmitted through the MMF is coupled into the cladding modes of the PCF due to the mode field mismatch between the MMF and the PCF. Similarly, at the PCF-SMF interface, the PCF cladding modes couple back into the core, thus creating interference with the PCF core mode which couples with the fundamental mode of the right SMF. The two collapsed regions at the PCF ends are due to the splicing of MMF-PCF and of PCF-SMF, resulting in a slightly tapered cladding of the PCF. The PCF is used as the sensing element of the MZI. The extended PCF length due to the applied strain induces a change in $\Delta n_{\mathrm{eff}}$ = $n_{\mathrm{eff},\mathrm{core}}$ − $n_{\mathrm{eff},\mathrm{cladding}}$, where $n_{\mathrm{eff},\mathrm{core}}$ and $n_{\mathrm{eff},\mathrm{cladding}}$ are the effective refractive indices of the PCF core and cladding modes, respectively. As a consequence of the $\Delta n_{\mathrm{eff}}$ variation, a wavelength shift of the MZI response can be observed. In Figure 8a, the experimental wavelength blue-shifts of the MZI transmission spectra as a function of different applied strains increasing from 0 to 5000 με is shown for four different lengths of the MMF section (PCF length: 40 mm). A strain sensitivity as high as −2.21 pm/με is obtained for a 20 mm-length MMF over a large measurement range. In Figure 8b, the wavelength blue-shifts of the MZI transmission spectra over the same measurement range is shown for four different lengths of the PCF section (MMF length: 30 mm). A strain sensitivity of −1.21 pm/με is obtained for a 40 mm-length PCF. The experimental measurements reported in Figure 8 show that the length of the MMF or PCF do not have a strong influence on the strain sensitivity [15]. Moreover, an interference fringe visibility of 24 dB is obtained [15].

**Figure 9:**
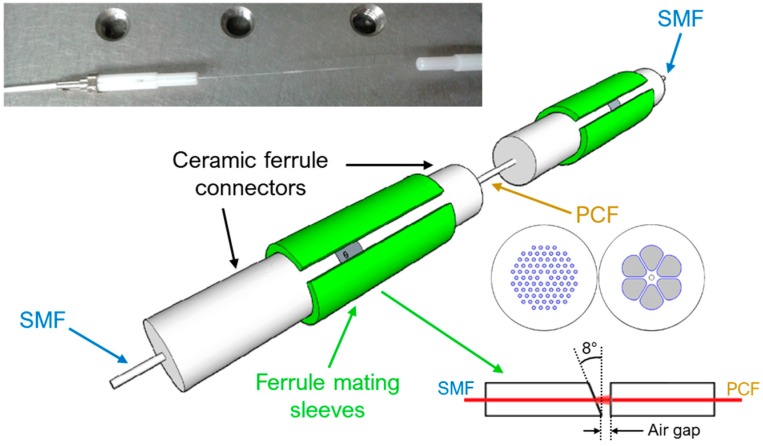
All-fiber Mach-Zehnder interferometer TNT gas sensor based on an in-line butt-coupled SMF-PCF-SMF structure. An image of the fabricated MZI device is also shown. Adapted from [16].

**Figure 10:**
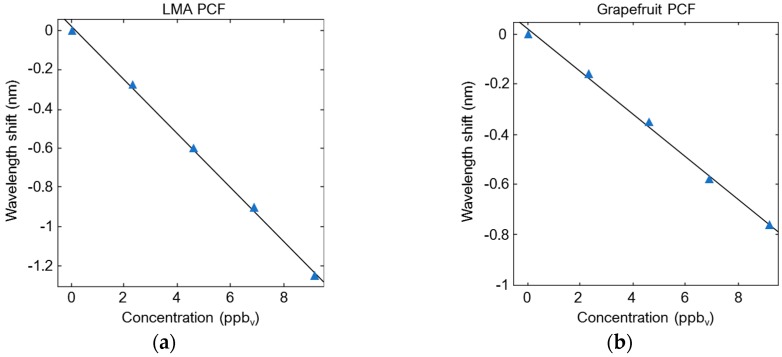
(**a**) Wavelength blue-shift of the interference dip centered at ~1550 nm due to the increasing TNT vapor concentrations. (**b**) Wavelength blue-shift of the interference dip centered at ~1553 nm due to the increasing TNT vapor concentrations. Adapted from [16].

In [16] two different kinds of SMF-PCF-SMF in-line Mach-Zehnder interferometers are fabricated and used as a gas sensor to detect the explosive trinitrotoluene (TNT) vapor. As illustrated in Figure 9, a large-mode-area (LMA) PCF and a grapefruit PCF are used to design and fabricate the two MZIs. The LMA PCF has a core diameter of 12 μm and a cladding with five layers of air holes surrounding the core (pitch Λ = 8.2 μm, air-hole diameter: 0.52Λ). The core and cladding are made of pure silica with a refractive index of 1.444. The grapefruit PCF has an outer cladding diameter of 125 μm, an inner cladding diameter of 16 μm and a core diameter of 6 μm. Between the inner and the outer claddings there are six large air holes with a diameter of 30 μm in the radial direction. The refractive indices of the germanium-doped core and the bulk silica glass surrounding the core are 1.479 and 1.457 at λ = 1.55 μm, respectively. The PCF is butt-coupled with the input and output SMFs to form a modal interferometer. Due to the butt coupling, two small air gaps at both ends of the PCF arise. The first air gap allows the excitation of certain cladding modes of the PCF, in addition to the core mode, via free space beams. The cladding modes couple back with the core when they meet the second air gap, thus creating interference between the core and cladding modes. The fiber ends forming each air gap are slid into ceramic ferrule connectors. To minimize back reflections, 8° pre-angled ceramic ferrules on each end of SMFs are used. Moreover, the cores of each terminated fiber end are aligned with the ceramic ferrule connectors by means of ferrule mating split sleeves. For proper coupling with the cladding modes of the PCF, air gaps approximately equal to 200 μm and 250 μm are used for LMA PCF and grapefruit PCF, respectively. The air gaps are also used as an inlet/outlet region for the gas. The physical length of both PCFs are optimized in terms of visibility of the interference fringes. For a 60 mm-long LMA PCF and a 60 mm-long grapefruit PCF, a high visibility of the interference fringes is observed. The MZI sensor is fabricated by depositing a 0.5–0.6 μm ployallylamine (PAH) film on the inner surface of the cladding air holes of the PCF. The PAH is used as a TNT recognition polymer layer which can selectively bind TNT molecules on the functionalized surface. As the TNT concentration increases, a blue-shift of the PAH-coated LMA PCF-MZI transmission spectra is observed. In Figure 10a the wavelength variation due to the increasing of TNT vapor concentration from 0 ppb_v_ (dry air) to 9.15 ppb_v_ (TNT-saturated air) is shown for the interference dip centered at ~1.55 μm, where ppb_v_ are parts per billion by volume. The wavelength blue-shift is nearly linear. A sensitivity of 140 pm/ppb_v_ and a limit of detection of 0.2 ppb_v_ (sensor resolution: 27 pm) are calculated. A blue-shift of the interference transmission spectra for increasing TNT concentrations is also observed for the PAH-coated grapefruit PCF-MZI sensor. As depicted in Figure 10b, the wavelength shift of the interference dip at ~1553 nm is linear over the entire TNT vapor concentration range. A sensitivity of 84 pm/ppb_v_ is achieved. A detection limit of 1.0 ppb_v_ (sensor resolution: 85 pm) is calculated, which is higher than the detection limit calculated in the previous case due to the lower Q-factor of the grapefruit PCF.

**Figure 11:**
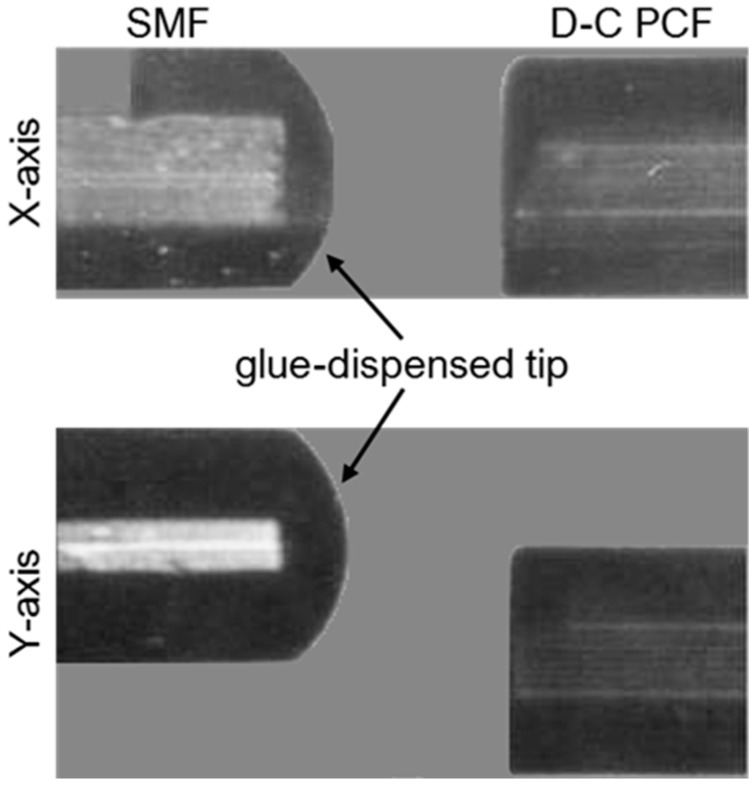
Microscope image of the optical alignment in the *X*- and *Y*-directions between the glue-dispensed SMF tip and the two-cores PCF tip. The offset in the Y-directions is used to excite higher order modes in the unblocked PCF core. Adapted from [18].

In [18] a temperature and strain sensor based on a partially liquid-filled dual-core photonic crystal fiber (D-C PCF) multi-components interferometer is experimentally investigated. In Figure 11a the cross-section of the D-C PCF is shown together with the optical alignment in the *X*- and *Y*-directions with the lead-in SMF. The D-C PCF used in the experiments has two solid cores located symmetrically with respect to the fiber center and an outer cladding with a diameter of 125 μm and 5 rings of circular air holes. The diameter of the air holes is about 3 μm and the hole pitch is 3.7 μm. The SMF was dip coated with a drop of glue at the fiber tip and, then, moved towards the D-C PCF along the X-axis by means of the stepping motor of a fusion splicer. The glue on the SMF tip is transferred to the D-C PCF tip so that the cladding air holes surrounding one of the PCF fiber cores are covered with the glue. Then, the partially blocked D-C PCF is immersed into a liquid having a thermal-optic coefficient equal to −3.41 × 10^−4^ RIU/°C. The liquid is used to fill the unblocked cladding air holes. After infiltration, the partially liquid-filled D-C PCF was spliced between the lead-in and lead-out SMF sections. An offset along the propagation direction is induced in one splicing point to excite higher order modes. As the ambient temperature changes, the refractive index of the filling liquid changes significantly due to the thermal-optic effect. Therefore, the fundamental modes propagating in the two cores undergo a phase change due to the difference between the effective mode refractive indices, resulting in a large interference fringe spectrum. Moreover, finer interference fringes can be observed due to the interference between the fundamental mode and the higher order modes of the glue-covered D-C PCF core [18].

**Figure 12:**
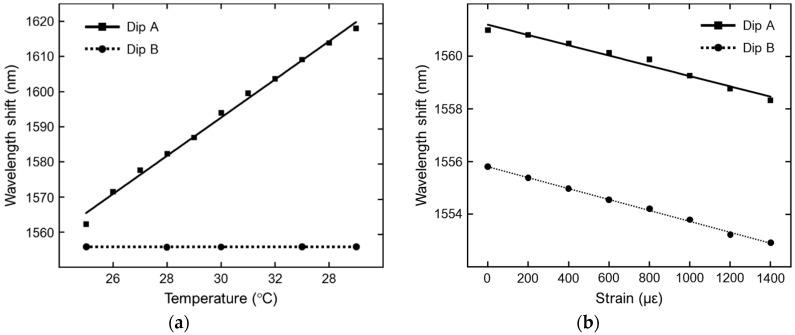
(**a**) Wavelength shifts of one dip of the larger interference fringes (dip A) and of one dip of the finer interference fringes (dip B) as a function of temperature. (**b**) Wavelength shifts of dip A and dip B as a function of strain. Adapted from [18].

In Figure 12a, the temperature sensitivity of the interferometer in terms of wavelengths shifts of the interference fringes is shown. The experimental results are obtained for a D-C PCF length of 6.8 cm exposed to a temperature gradually increasing from 25 °C to 35 °C. Dip A refers to one dip of the spectrum envelope of the larger interference fringes, while Dip B is one dip of the finer interference fringes. Dip B undergo a relatively small red-shift, the sensitivity being equal to 0.012 nm/°C. Dip A exhibits a huge red-shift, with a sensitivity equal to 5.43 nm/°C, due to the high thermal-optic coefficient of the RI liquid surrounding the temperature-sensitive core of the PCF. In Figure 12b, the strain sensitivity of the same interferometer in terms of wavelengths shifts of the interference spectra is shown, the applied strain ranging from 0 to 1400 με. In this case, the dip A of the larger interference fringes and the dip B of the finer interference fringes exhibit almost the same blue-shift, the sensitivity values being equal to −1.95 pm/με and −2.08 pm/με, respectively. Simultaneous measurements of strain and temperature can be achieved by using the standard matrix demodulation method [18].

## 4. Fiber Bragg Grating-Based Sensors

A fiber Bragg grating (FBG) is an optical structure in which the refractive index of the fiber core exhibits a periodic variation. This refractive index modulation causes light of a suitable wavelength, called Bragg wavelength λ_B_, to be reflected by means of Fresnel reflection. Hence, the grating acts like a wavelength-selective mirror. The Bragg wavelength of the grating is closely related to its period Λ. The number of periodic variations is also important since it determines the selectivity of the grating.

Fiber Bragg gratings can be investigated by means of the coupled mode theory. In terms of optical modes, the FBG allows the coupling of different modes, both co-propagating and counter-propagating, therefore transferring the optical power from one mode to another. In particular, short-period gratings (SPG) and long-period gratings (LPG) are employed to transfer power to counter-propagating and co-propagating optical modes, respectively. Cladding modes can be excited too, thus enabling the realization of versatile optical sensing systems [20,21,22,23,24,25,26,27,28]. There are many types of structures which can be used to make a fiber Bragg grating: (i) uniform FBG, in which there is a uniform positive-only index change, (ii) chirped FBG, in which the grating period is varied linearly, (iii) tilted FBG, in which the variation of the refractive index is at an angle with respect to the optical axis, (iv) superstructure FBG, in which several small FBGs are placed close to one another, (v) apodized FBG, which exploits the grading of the refractive index.

**Figure 13:**
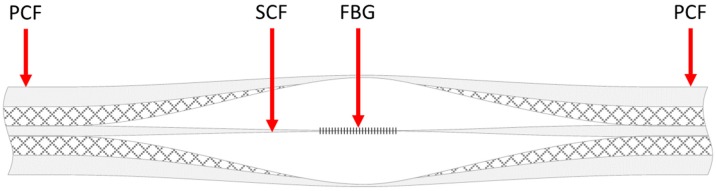
Scheme of the FBG-embedded PCF sensor fabricated by means of selective inflation. Adapted from [20].

**Figure 14:**
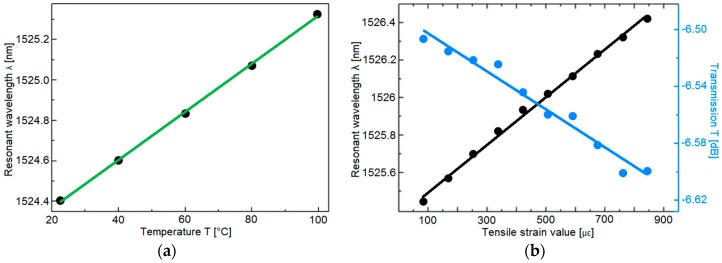
Measured wavelength shift of the FBG-embedded PCF sensor as a function of (**a**) temperature and (**b**) axial tensile strain. Adapted from [20].

In [20] an FBG sensor based on a selectively inflated photonic crystal fiber (PCF) is fabricated. Figure 13 shows the schematic of the proposed sensor. Starting from a PCF, the selectively inflation technique makes it possible to obtain a region with a suspended-core fiber (SCF) structure, suitable for the inscription of the FBG. The SCF core has a diameter of about 4.5 µm, while the width of the 3 struts is about 400 nm. An 800 nm Ti:sapphire laser emitting 100 fs pulses with a repetition rate of 1 kHz is employed to inscribe the FBG. Beam focusing is achieved by means of a cylindrical lens, whose focal length is 50 mm. The measured Bragg wavelength of the inscribed FBG is 1528 nm, with a full width at half maximum (FWHM) of about 1 nm. The FBG-embedded PCF can be exploited for the sensing of temperature, strain and even refractive index. Moreover, the simple structure of the SCF makes it possible to open windows on the fiber in order to develop microfluidic sensing systems. Figure 14a reports the measured resonant wavelength as a function of the temperature, in the range 22.6–99.9 °C. As the temperature increases, the resonant wavelength undergoes a red shift. A linear fitting between the resonant wavelength and the temperature is used to calculate the sensitivity of the sensor, which turns out to be 11.9 pm/°C. Figure 14b shows the measured resonant wavelength as a function of the axial tensile strain, in the range 80–850 µε. In this case, along with a red shift in the resonant wavelength, a variation in the intensity of the dip is also observed. The calculated sensitivities with respect to the axial tensile strain are 1.27 pm/µε for the resonant wavelength and 1.33 dB/µε for the dip intensity.

**Figure 15:**
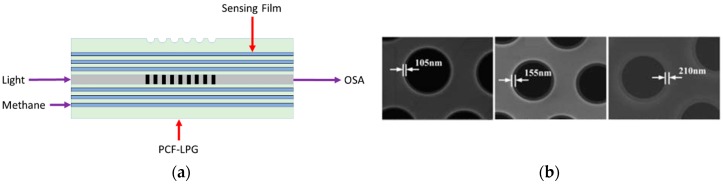
(**a**) Schematic diagram of the PCF-LPG methane sensor; (**b**) SEM image of the PCF-LPG cross-section with the cladding air holes coated with the PAA-CNTs/PAH/cryptophane-A-6Me sensing film having different thickness, 105 nm, 155 nm, and 210 nm, respectively. Adapted from [21].

Gas sensing is a hot topic for a number of applications in industry, medicine, safety and risk control, environment monitoring. In [21] a methane sensor based on a long-period grating inscribed in a photonic crystal fiber (PCF-LPG) is experimentally investigated. In Figure 15a the schematic diagram of the PCF-LPG methane sensor is illustrated. To increase the PCF-LPG sensitivity to methane detection, the inner surface of PCF cladding air holes is coated with a negatively charged poly(acrylic acid)-carbon nanotubes (PAA-CNTs) film alternately attached to a positively charged polypropylene amine hydrochloride (PAH) film by means of an electrostatic self-assembly technique. Then, the cryptophane-A-6Me molecules are absorbed by the PAA-CNTs/PAH nanofilms to form the methane sensing film. Three PCF-LPG methane sensors with different thickness of the sensing film, 105 nm, 155 nm, and 210 nm, are fabricated (Figure 15b). 

**Figure 16:**
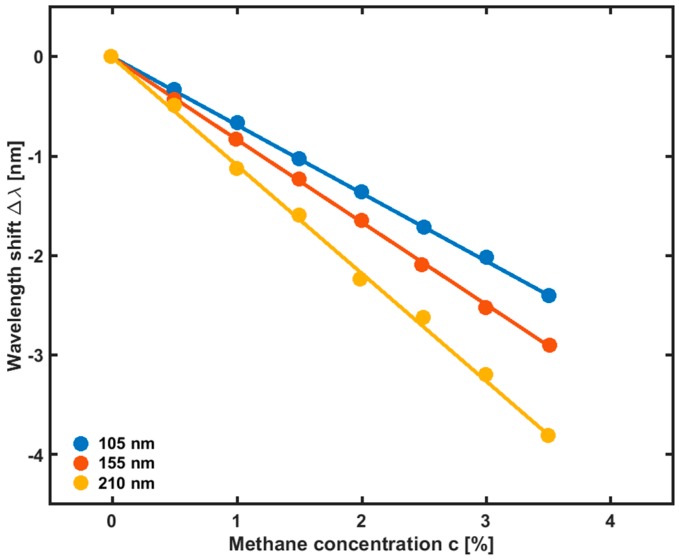
Wavelength blue-shift of the PCF-LPG methane sensor as a function of the increasing methane volume concentration for sensing film thicknesses of 105 nm, 155 nm, and 210 nm, respectively. Adapted from [21].

The refractive index (RI) of the sensing film, n, is measured in the 0.0–3.5% range of the methane volume concentration, c. The measurements show that the film RI linearly decreases as the methane concentration increase. In Figure 16 the wavelength shift of the PCF-LPG sensor response as a function of the methane concentration is shown for the three aforementioned sensing film thicknesses. As the film thickness increases from 105 nm to 210 nm, the resonant wavelength undergoes a blue shift. For the film thickness of 210 nm, the sensitivity S is 1.078 nm/%, with a detection limit of 0.18%. The resonant wavelength shifts from 1545.80 nm to 1542.00 nm as the methane concentration increases from 0.0% to 3.5% (v/v). Moreover, it has been experimentally shown that the PCF-LPG sensor is insensitive to other common gases, such as N_2_, O_2_, CO, CO_2_, and H_2_S, and that the influence of temperature and humidity fluctuations on the sensor response is not significant [21].

**Figure 17:**
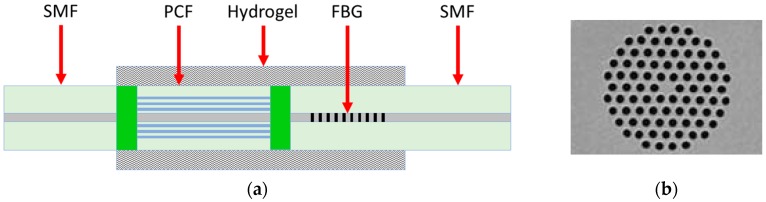
(**a**) Schematic diagram of the hydrogel-coated PCF-FBG sensor; (**b**) SEM image of the cross-section of the PCF. Adapted from [22].

**Figure 18:**
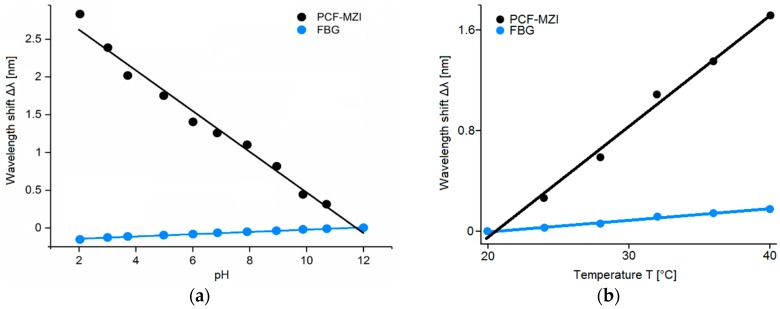
(**a**) Wavelength shifts of the PCF-FBG sensor response for pH values in the range 2–12 at a fixed temperature of 22 °C; (**b**) Wavelength shifts of the PCF-FBG sensor response for temperature variations in the range 20–40 °C at a fixed pH value of 10. Adapted from [22].

In [22] the fabrication and the experimental characterization of a pH and temperature sensor based on an in-line PCF-based Mach-Zehnder interferometer with an FBG is illustrated. The schematic diagram of the PCF-FBG sensor is shown in Figure 17a. Figure 17b shows a SEM image of the PCF cross-section. The PCF-FBG sensor consists of a photonic crystal fiber section spliced between two standard single-mode fiber sections. The FBG is inscribed in the lead-out SMF. The lengths of the PCF and the FBG used in the experiment are 20 mm and 10 mm, respectively. To effectively monitor variations of the ambient pH and temperature values, the surface of the sensor covering the PCF and the FBG is functionalized with a pH-sensitive hydrogel coating. As the pH value and/or temperature changes, the hydrogel layer undergoes a swelling or shrinking inducing the variation of its refractive index and, therefore, the variation of the effective refractive index of the PCF cladding modes. As a result, a wavelength shift in the transmission spectrum of the interference between core and cladding modes can be observed. At the same time, the swelling or shrinking of the hydrogel layer induces a strain effect on the FBG, thereby changing the grating period and inducing a shift of the Bragg wavelength. In this way, the PCF-FBG structure is simultaneously sensitive to ambient pH and temperature fluctuations. To study the pH response characteristics, the PCF-FBG sensor is immersed into a solution with pH values increasing from 2 to 12 (causing the swelling of the hydrogel). To exclude the influence of the temperature variation, the process is held at 22 °C. are 0.088 nm/°C and 0.009 nm/°C, respectively, as depicted in Figure 18b. The temperature detection limit is 0.2 °C, due to the same OSA resolution (0.02 nm). The simultaneous measurement of pH and temperature can be achieved by calculating a sensitivity matrix with the sensitivity coefficients of the PCF and FBG to the temperature and pH.

The interference spectrum due to the PCF region undergoes a blue shift as the pH value increases. The swelling of the hydrogel layer causes the strain of the FBG region, which in turn leads to a red shift of Bragg wavelength. These different behaviors are shown in Figure 18a. The pH sensitivities for the PCF and the FBG are −0.271 nm/pH and 0.015 nm/pH, respectively. The pH detection limit is about 0.1 pH due to the 0.02 nm resolution of the OSA used in the experiment. To study the temperature response characteristics, the PCF-FBG sensor is immersed into a solution with a fixed pH value of 10, while the temperature is increased from 20 °C to 40 °C. The temperature sensitivities for the PCF and the FBG.

The use of a suitable long period grating LPG to obtain the interference between the core and the cladding modes and the use of a rare earth allowing lasing has been exploited in [29], where an accurate design of a fiber optic temperature sensor is proposed. In particular, the proposed sensor is based on a cascade of three PCFs. The first PCF includes a suitable cascade of long period gratings, designed into the core. Peculiar inner cladding modes are coupled with the fundamental core mode at the pump wavelength via the grating cascade as described in [30]. The other two PCF sections are a single mode intermediate and a rare-earth activated Fabry-Perot optical cavity. The main idea is that the variation of the temperature affects the pump coupling. The feasibility investigation has been performed by developing a computer code based on the rate equations and power propagation equations in rare earth doped fibers, well validated and used for the investigation of other active devices [31,32]. The simulations promise a complete set-up for temperature monitoring which could be obtained by utilizing only a low-cost pump diode laser at 980 nm wavelength and a commercial optical power detector. The simulated sensitivity S = 315.1 μW/°C and the operation range ΔT = 100 °C is of interest for actual applications.

## 5. Coated PCF Sensors

**Figure 19:**
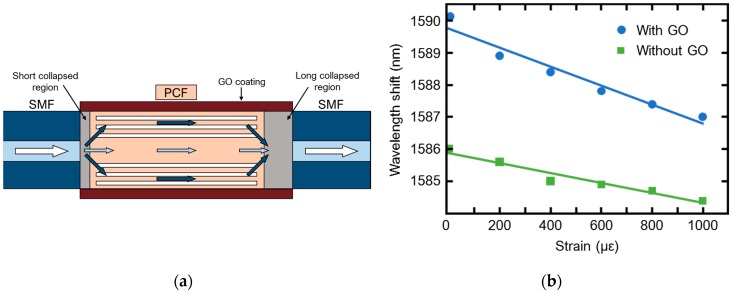
GO coated PCF sensor; (**a**) Sketch of the operation principle; (**b**) strain sensitivity of the GO coated and uncoated SMF-PCF-SMF sensor. Adapted from [38].

In addition to the metal coating employed to obtain plasmonic PCFs, a variety of other coatings can be employed for fiber sensing. In particular, graphene has been used for this purpose due to its optical and electronic properties [33]. Graphene layers/films are characterized by a two-dimensional carbon material with one-atom-thickness. It is a very promising candidate for several applications as the fabrication of innovative field effect transistors and transparent conductive films. A drawback of this material is related to the difficulty to produce high-quality graphene in large quantity. Therefore, the use of graphene oxide (GO) instead of graphene is considered a good trade-off between properties of graphene characteristics and the synthesis cost/difficulty. A wide range of GO chemical sensors, biosensors and gas sensors have been proposed [34,35,36,37,38,39]. In [38] a GO coated photonic crystal fiber (PCF) has been fabricated to obtain a modal Mach-Zehnder interferometer for strain and temperature sensing. The interferometer probe was obtained by splicing a PCF between to single mode fiber (SMF) terminations, see Figure 19a. The splicing of the cascade SMF-PCF-SMF has been performed asymmetrically, in order to create a suitable short collapsed region at the first SMF-PCF junction and a long-collapsed region at the second junction. The collapsed regions have been coated with GO. The short-collapsed region has been used as an input section, to excite core and cladding modes in PCF. The Mach-Zehnder interferometer behaviour is obtained thank to the difference in phase constant between core and cladding modes. During their propagation, the evanescent wave interacts with the GO coating, especially in long collapsed region. The change in refractive index of GO allows to monitor strain. The improvement due to the use of GO has been demonstrated since the uncoated probe has shown a strain sensitivity of 1.6 pm/με while the GO coated probe has shown a strain sensitivity of 3.1 pm/με, as illustrated in Figure 19b. This value is 93% higher than the same probe without GO coating. The temperature sensitivity is of the order of 14 pm/°C. Moreover, both the strain and temperature response has exhibited a linear behaviour. The obtained results prove that in line of principle the use GO coating can enhance the PCF sensor behaviour.

**Figure 20:**
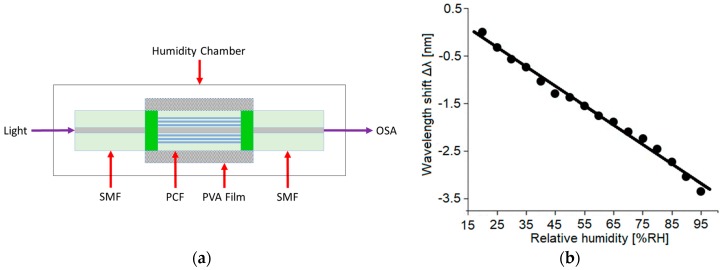
PVA coated SMF-PCF-SMF sensor; (**a**) Sketch of the experimental setup and operation principle of the humidity; (**b**) Measured wavelength shift as a function of the relative humidity. Adapted from [41].

Moreover, graphene can be employed to bio-functionalize suitable PCFs, e.g., for the detection of Dengue virus (DENV) IIE proteins [39]. Other materials employed for PCF coating are similarly promising. As an example, polyvinyl alcohol (PVA) [40,41] and ferrofluid [42] coatings have been successfully applied. In [41] a PVA coated PCF has been proposed and experimentally demonstrated to obtain a relative humidity (RH) sensor. As in several previous cases, the sensor is obtained by fusion splicing of a short length of PCF between two single-mode fibers. The sketch of the sensing set-up is shown in Figure 20. 

The spliced PCF is fully collapsed and then coated with a layer of PVA by using a dip-coating process. Both core and cladding modes are excited in the collapsed fiber section. The two propagating modes recombine in the second fusion splicing region thus they interact as in a Mach-Zehnder interferometer. Interesting sensing characteristics, good repeatability and low temperature independence have been obtained. The fabricated sensor has exhibited a humidity sensitivity of 40.9 pm/% RH within a measurement range of 20–95% RH.

In [42] a compact magnetic field sensor based on a tapered photonic crystal fiber (PCF) coated with ferrofluid has been reported. A tapered PCF with a waist diameter of 24 μm has been spliced between two SMF, by obtaining the well-known SMF-PCF-SMS optical structure. A water-based ferrofluid (EMG507, Ferrotec, Shanghai, China) with a particle volume concentration of 1.8% has been used in the experiment. The Fe_3_O_4_ nanoparticles of ferrofluid water solution have nominal sizes of 10nm. The ferrofluid solution has been filled in a capillary to coat the PCF taper. The sensitivity of the ferrofluid coated PCF sensor is close to 4 × 10^−5^ RIU/Gs and the RI sensitivity in term of wavelength shift of the evanescent field is 401 nm/RIU. Therefore, magnetic field can be efficiently sensed. By increasing the magnetic field H from 100 to 600 Gs the modal interference spectrum is shifted with a sensitivity of 16.04 pm/Gs. Another interesting ferrofluid coated PCF sensor has been proposed in [43]. In this last case a coated offset fusion splice is considered instead of the coated taper in order to obtain the suitable mode interaction to be exploited for sensing. However, many other materials can be employed in order to functionalize the PCFs or in order to simply improve the evanescent field interaction with the analyte. As an example, polymeric sensitive layers of Polydimethylsiloxane (PDMS) can be employed for detection of toluene and chlorobenzene in water/air or methadone in biological fluids [44,45,46]. At equilibrium, the hydrocarbon contaminant concentration in the PDMS and in the water are linked by a peculiar distribution constant. The analyte can have a concentration hundreds of times larger in PDMS if compared with that in water. Moreover, the PDMS is water repellent and has an absorption loss which increases by increasing the contaminant concentration. A change of the imaginary part of the complex effective refractive index of the guided modes allow the detection. In particular, the light guided in the fiber core suffers a power attenuation due to chlorobenzene, toluene, methadone or other hydrocarbons. As an example, the simulated absorbance sensitivity for a fiber length L = 10 cm having a microstructured exposed core fiber without PDMS is close to A = 0.3 × 10^−6^ ppb^−1^, while it is close to A = 0.011 ppb^−1^ for exposed core fiber with PDMS [46]. It is well known that the PCF sensible region can be close to the core or in a suitable internal region. This is an alternative strategy with respect to consider as sensible region the cladding or the external coating. As an example, several papers report hollow core fibers filled or partially filled by gas and liquids to be detected [47,48,49]. In [49] the sensor is liquid-filled hollow core fiber spliced between single mode fibers. A temperature sensitivity of −42.7 pm/°C has been experimentally obtained. The wavelength shift response to the ambient liquid RI exhibits a sensitivity of 141 nm/RIU. Moreover, it is possible to monitoring environment temperature and RI variation simultaneously.

## 6. Conclusions

Recent PCF sensors based on plasmon resonance, interferometry, gratings, sensitive materials covering the cladding or the hollow core have been briefly illustrated. More precisely, significant examples of PCF sensors for the measurement of immunoglobulin G, refractive index, strain, temperature, hydrogen concentration, gases as methane or vapor of the explosive trinitrotoluene (TNT), magnetic field, have been concisely discussed. Table 1 collects the main characteristics of these devices; they are listed by considering the sensing region and/or operation peculiarity. The high performances reported in the last four columns of the table show the PCF sensor potential, promising ever increasing applications in a variety of areas. The experimental results reported in this review and summarized in Table 1 clearly show that recent PCF sensors tend to exploit different sensing operational principles at the same time (e.g., coating/interferometry/grating, coating/SPR and so on) in order to increase the sensitivity. 

In particular, handling with high control the dispersion and waveguiding properties of the microstructured core and inner cladding regions allow the maximization of evanescent field based sensing and the integration of the interferometric geometry. Further applications will be possible in the next future thank to the recent advances on glasses/optical fibers operating in MiD-IR [50,51,52] where several biomolecules and polluting contaminants exhibit their spectral fingerprint and the development of novel PCF sensors and lasers are expected with the aim to extend the operation wavelength range of devices for biomedicine application [14,22,39,53].

## Figures and Tables

**Table 1:** Comparison of photonic crystal fiber sensors.

Ref.	Type	Sensing Region	Detection Type	Range	Sensitivity	Resolution	R^2^
[3]	SPR PCF	Gold nanowire	Refractive Index	1.33–1.36 RIU	5933 nm/RIU	2.81 × 10^−6^ RIU	0.98388
[6]	SPR Twin Core PCF	Gold film	Refractive Index	1.33–1.40 RIU	4000 nm/RIU	1.11 × 10^−5^ RIU	0.9784
[10]	SPR D-shaped PCF	Gold film	Refractive Index	1.33–1.43 RIU	9800 nm/RIU	2.2 × 10^−6^ RIU	-
[12]	SPR MMF-PCF-MMF	Gold/PDMS film	Refractive Index	1.33–1.39 RIU	-	-	0.9987
			Temperature	35–100 °C	−1.551 nm/°C	-	0.9977
[14]	SPR MMF-PCF-MMF	Protein A/Au nanoparticles/Au film	Biomolecules Concentration	1–15 μg/mL	0.53967 nm/(μg/mL)	37 ng/mL	0.9972
[15]	SMF-MMF-PCF-SMF in-line MZI	Collapsed region	Strain	0–5000 µε	−2.21 pm/µε	-	0.9875
[16]	PAH-coated LMA PCF-MZI	PAH	TNT vapor	0–9.15 ppb_v_	140 pm/ppb_v_	0.2 ppb_v_	-
[16]	PAH-coated grapefruit PCF-MZI	PAH	TNT vapor	0–9.15 ppb_v_	84 pm/ppb_v_	1.0 ppb_v_	
[18]	Dual Core PCF interferometer	Off-set splicing	Temperature	25–35 °C	5.43 nm/°C	-	-
			Strain	0–1400 µε	−2.08 pm/µε	-	-
[20]	FBG-embedded PCF	Grating	Temperature	22.6–99.9 °C	11.9 pm/°C	-	0.99924
			Strain	80–850 µε	1.27 pm/µε	-	0.9965
[21]	PCF-LPG	PAA-CNTs/PAH nanofilms -grating	Methane Gas	0.0–3.5% (v/v)	1.078 nm/%	0.18%	-
[22]	In-line PCF-MZI with FBG	pH-sensitive hydrogel	pH	2–12	−0.271 nm/pH	0.1 pH	0.989
			Temperature	20–40 °C	0.088 nm/°C	0.2 °C	0.993
[38]	GO-coated PCF-MZI	GO/splice	Strain	0–1000 με	3.1 pm/με	-	0.94
			Temperature	-	14 pm/°C	-	-
[41]	PVA coated PCF-MZI	Collapsed region/PVA	Relative Humidity	20–95% RH	40.9 pm/% RH	-	-
[42]	Ferrofluid-coated tapered PCF-MI	Fiber taper	Magnetic Field	100 to 600 Gs	16.04 pm/Gs	-	-
